# Obituary

**Published:** 1909-03-13

**Authors:** 


### OBITUARY.

THE death occurred last week at Ventnor of Dr. T. Easton, who was Chairman of the Southampton Liberal Unionist Association and prominently associated with numerous local educational institutions. Dr. Easton received his medical education at Edinburgh and St. Thomas's Hospital, London, and graduated in arts and medicine at the University of Edinburgh in 1884. He also studied at Berlin, and subsequently obtained the M.D. degree of his own University. He was medical officer to the Southampton Corporation.

MR. SAMUEL LEE RYMER, J.P., L.D.S., R.C.S.Eng., who died at Croydon on March 7, was formerly a prominent member of the dental profession. In 1856 he called a meeting of dentists at the London Tavern, Bishopsgate, and this was the first step towards the establishment of the dental diploma of L.D.S., R.C.S., and the foundation of the British Dental Association, of which body he was president in 1887. Mr. Rymer was an alderman and a justice of the peace, and was Mayor of Croydon in 1893, and one of the founders of the Croydon General Hospital.

